# Uptake of family planning methods among adults presenting with genital ulcers at community posts and public outpatient health facilities in central Uganda

**DOI:** 10.1186/s40834-026-00456-w

**Published:** 2026-05-09

**Authors:** Brenda Mary Dawa, Rosalind Parkes-Ratanshi, Annet Onzia, Emmanuel Mande, George Katende, Yukari C. Manabe, Matthew M. Hamill

**Affiliations:** 1 Infectious Diseases Institute, College of Health Sciences, Makerere University, P.O.BOX 22418, Kampala, Uganda; 2 Centre for Public Health, Queen’s University Belfast, Belfast, UK; 3 Division of Infectious Diseases, Johns Hopkins University School of Medicine, Baltimore, MD USA

**Keywords:** Family planning, Contraceptives, Genital ulcers, Sexually transmitted infections, Sexual heath, HIV, Uganda

## Abstract

**Background:**

Genital ulcer disease (GUD), a common clinical presentation at health facilities in Uganda, is often caused by sexually transmitted infections (STIs) and increases the risk of HIV. Family planning (FP) methods are effective for preventing unintended pregnancies, while some methods, namely male and female condoms, also reduce the risk of STI transmission. However, little has been documented about FP use among individuals with GUD in Uganda.

**Methods:**

Patients with clinician-confirmed genital ulcers were enrolled for a GUD study at six sites across government clinics and community outreaches in Kampala, Wakiso, and Kalangala, Uganda, between July/2023 and June/2024. Demographic, behavioral, and family planning use data were collected in English and Luganda using prespecified questionnaires. Urine and genital swabs were collected for gonorrhea and chlamydia testing and blood for HIV and syphilis antibodies using rapid diagnostic tests (RDT). Pregnant women were excluded from the analysis. Associations with FP use were explored using rank sum tests, chi-square, and Fisher’s exact tests as appropriate.

**Results:**

Of 104 participants with confirmed genital ulceration, 75(72.1%) were women, 12(16.0%) of whom were pregnant. Overall, of 92 non-pregnant participants, 36(39.1%) had a reactive HIV RDT; 10(27.8%) were new diagnoses. Nine (9.8%) had positive tests for gonorrhea, chlamydia, or syphilis. No men reported condom use. Of the 63 non-pregnant female participants analyzed, 29(46.0%) reported current FP use. Subdermal implants were the most used method in 15/29(51.7%). Twenty-one (33.3%) reported ≥ 2 sexual partners in the past 3 months. FP use was significantly associated with younger age, being married, transactional sex, ≥ 2 sexual partners in the past 3 months, and being sexually active in the past month.

**Conclusion:**

More than one-sixth of women with GUD were pregnant. The uptake of FP among individuals with GUD was low, and HIV/STI prevalence was high. Effective FP has a key role in preventing unintended pregnancies and mother-to-child transmission of STIs. These findings highlight the need for educational and policy strategies to increase sensitization on sexual health, including increasing uptake of FP and encouraging male condom use in a population at high risk of STIs.

**Supplementary Information:**

The online version contains supplementary material available at 10.1186/s40834-026-00456-w.

## Introduction

The total fertility rate (TFR), the number of children born per woman, has been declining for decades [1]; the smallest declines occurred in Sub-Saharan Africa (SSA) [2], with a fertility rate of 4.5 [3]. Uganda’s TFR of 4.7 is among the top 20 globally [4].

A cornerstone of maternal health, family planning (FP) is codified in Sustainable Development Goal 3.7, which aims to achieve “universal access to sexual and reproductive health-care services, including family planning, information and education, and integration of reproductive health into national strategies and programs [5].” In 2022, the global family planning prevalence of any method was estimated at 65%, and of modern methods at 58.7% for married or in-union women [6]. Uganda faces significant challenges in meeting family planning needs; recent data revealed the use of modern FP among married women was 38%, with 22% experiencing unmet FP needs [7].

Modern contraceptives include male and female sterilization, male and female condoms, depot medroxyprogesterone acetate (DMPA), subdermal implants (SDI), oral contraceptives, lactational amenorrhea, intrauterine devices (IUD), and emergency contraception [8]. While FP is a key strategy in preventing unintended pregnancies, only condoms are effective at preventing both pregnancy and sexually transmitted infections (STI), including HIV [6]. The use of male condoms for dual protection was reported in 35–55% of adolescents in SSA, including Uganda [9]. Women with HIV in SSA face gaps in access to sexual and reproductive health services, with only 20–43% using contraception, despite 67–92% wanting to limit childbearing [10]. Reducing the unmet need for contraception in women living with STIs, including HIV, can not only improve reproductive health outcomes but also reduce the risk of mother-to-child transmission [11].

STIs that cause genital ulcer disease (GUD), such as syphilis, Mpox, and herpes simplex virus [12], can cause preterm delivery, fetal demise, and serious morbidity in newborns [13, 14]. Syphilis rates in pregnant women in Uganda are high; in one study in Kampala in 2019–2020, syphilis prevalence was 5.9% [15]. Despite STIs being a marker of high-risk sexual behaviors, FP choices in people with GUD in Uganda have not been previously evaluated. We assessed current FP use in adults with clinician-confirmed genital ulcer disease in three districts in central Uganda.

## Methods

### Study setting

An observational cohort study assessing the epidemiology and etiology of GUD was conducted from July 2023 to June 2024 at three government health centers and three community health posts in Kampala, Wakiso, and Kalangala, Uganda [16]. Data from the baseline study visit are presented.

### Study population

Potential participants, men and women aged 18 years and above, attending health facilities seeking care for GUD symptoms were referred to the study team after completion of their clinical care visit, which included syndromic management of STIs per Ugandan treatment guidelines [17]. Additionally, we reached those who were not actively seeking health care at health centers through community mobilizations by village health teams (VHT) [18, 19]. VHTs were located in urban slums in Kampala and Wakiso and on the Kalangala islands.

### Study procedures

After study initiation at the clinical sites, the study team sensitized clinicians to inform potentially eligible patients about the study and to refer them to the study team. In community venues, VHTs were asked to mobilize their communities; they actively engaged residents through home visits and private indoor discussions while discreetly identifying individuals with signs and symptoms of STIs and GUD. Potential participants were advised that they could go to a particular community post on the designated day to meet the study team. After consent, a physical examination was performed by a research clinician to confirm the presence and nature of genital ulceration prior to enrollment. Those who did not have GUD, had taken antibiotics with anti-treponemal activity in the past 28 days, were not willing to provide samples for STI testing, or who were deemed too ill were excluded. Participants were screened and enrolled consecutively after they were seen by a clinician at the health facility or registered their interest in participation with the VHT.

After enrollment, demographic, socio-behavioral, and clinical data were collected using interviewer-administered questionnaires. Data were captured using the REDCap mobile application (Vanderbilt University, Tennessee, USA), which were checked weekly by the study data manager for completeness to ensure quality. Sociodemographic characteristics included age, sex, marital status, level of education, employment status, recruitment site (clinic vs. community), and alcohol and drug use. Sexual behavior data included sexual activity in the past one month, any involvement in transactional sex in the past 6 months, and the number of sex partners in the past 3 months. Clinical data included the type of FP used at the time of enrollment: men were asked about condom use and vasectomy; women were asked about DMPA injectables, oral contraceptives, SDI, emergency contraception, condoms, IUD, and tubal ligation. STI testing and treatment history were also collected.

Using standard venipuncture techniques, study clinicians collected blood for HIV and syphilis antibody rapid diagnostic tests (SD Bioline duo, Abbot Diagnostics, Princeton, NJ); the results were returned to the participants on the day of recruitment. Self-collected genital swabs for women and urine for men were transported on the same day to the Translational Research Laboratory at the Infectious Diseases Institute (IDI) and tested for *Neisseria gonorrhoeae* (*Ng*) and *Chlamydia trachomatis* (*Ct*) using nucleic acid amplification tests (Xpert, Cepheid, Sunnyvale, CA, USA). Test results were issued within one working day following receipt.

Participants who tested positive for treponemal antibodies, *Ng*, or *Ct* were treated according to Ugandan national treatment guidelines [17] by the study team unless appropriate treatment had already been provided at the health facilities as part of syndromic management. Participants newly identified with HIV were referred to the nearest health facility for confirmatory laboratory testing and initiation of antiretroviral therapy (ART).

### Statistical analysis

FP methods used by the female partners of male participants were not assessed; therefore, the main analysis was restricted to female participants. Sociodemographic, clinical, and behavioral characteristics were described for all female participants and by FP status (current use vs. no use) using frequencies and proportions or medians and interquartile ranges (IQRs) for categorical or numeric data, respectively. Group differences by FP use status were compared using rank sum test, chi-square, and Fisher’s exact test as appropriate. P-values ≤ 0.05 were considered statistically significant. Data were analyzed using STATA version 17.0 (College Station, Texas, USA).

### Ethical oversight

In Uganda, the IDI Research Ethics Committee (IDI-REC-2022-26) and the Uganda National Council for Science and Technology (HS2728ES) approved the study. Approval was also obtained from the Johns Hopkins University Institutional Review Board (IRB:00376680). No study procedures were commenced until written informed consent had been obtained either in Luganda or English per participant preference. Participants were not compensated for joining the study but were provided with 40,000 UGX (~ 11 USD) for transportation. No costs for testing, treatment, or referral were incurred by participants.

## Results

### Sociodemographic characteristics of participants

In total, 104 participants were enrolled; 72.1% (75 of 104) were women, of whom 16.0% (12 of 75) were pregnant; data from 92 participants (of whom 68.5% (63) were non-pregnant women) were analyzed. Two thirds (63.5%) were enrolled from community, non-clinical sites. Baseline socio-demographic characteristics, sexual behaviors, methods of FP, and results of diagnostic tests for the female participants are presented in Table 1. The median (IQR) age was 29(24-38) years; 31.7% were < 25 years old. All women who provided responses were less than 53 years old; none were post-menopausal.

**Table 1:** Baseline characteristics and Family Planning Uptake in Ugandan women with Genital Ulcer Disease

Variable	All *n* = 63 (100.0) *n*(col%)	Family Planning Use		*P*-value
		Yes *n* = 29 (46.0) *n* (col%)	No *n* = 34 (54.0) *n* (col%)	
**Age** (years) Median (IQR)	29 (24–38)	26 (22–30)	33 (27–41)	**0.002** ^**a**^
**Age range** (years)				**0.024**
≤ 24	20 (31.7)	14 (48.3)	6 (17.6)	
25–34	22 (34.9)	9 (31.0)	13 (38.2)	
≥ 35	21 (33.3)	6 (20.7)	15 (44.1)	
**Marital status**				**0.035** ^**c**^
Single, never married	17 (27.0)	11 (37.9)	6 (17.6)	
Married, cohabiting	31 (49.2)	15 (51.7)	16 (47.1)	
Separate, divorced & widowed	15 (23.8)	3 (10.3)	12 (35.3)	
**Study site**				0.060
Clinic	23 (36.5)	7 (24.1)	16 (47.1)	
Community	40 (63.5)	22 (75.9)	18 (52.9)	
**HIV status (Self-reported)**				0.643^c^
Positive	19 (30.2)	8 (27.6)	11 (32.3)	
Negative	35 (55.6)	18 (62.1)	17 (50.0)	
Unsure	9 (14.3)	3 (10.3)	6 (17.6)	
**Highest level of education**				0.765^c^
< Primary seven	25 (39.7)	12 (41.4)	13 (38.2)	
≥Primary seven	35 (55.5)	15 (51.7)	20 (58.8)	
None	3 (4.8)	2 (6.9)	1 (2.9)	
**Employment**				0.214
Employed	44 (69.8)	18 (62.1)	26 (76.5)	
Unemployed	19 (30.2)	11 (37.9)	8 (23.5)	
**Family planning method used** ^**b**^				na
Condom use	3 (10.3)	3 (10.3)	na	
Injections (DMPA)	8 (27.6)	8 (27.6)	na	
Subdermal implant (SDI)	15 (51.7)	15 (51.7)	na	
Coil or IUD	3 (10.3)	3 (10.3)	na	
**Transactional sex** (in past 6 M)				**0.002** ^**c**^
Yes	18 (28.6)	14 (48.3)	4 (11.8)	
No	45 (71.4)	15 (51.7)	30 (88.2)	
**Number sexual partners** (in past 3 M)				**0.026** ^**c**^
None	6 (9.5)	0 (0%)	6 (17.6)	
1	36 (57.1)	16 (55.2)	20 (58.8)	
≥ 2	21 (33.3)	13 (44.8)	8 (23.5)	
**Alcohol use** (in past 3 M)				0.946
Yes	22 (34.9)	10 (34.5)	12 (35.3)	
No	41 (65.1)	19 (65.5)	22 (64.7)	
**Previously tested for an STI including HIV**				0.770^c^
Yes	6 (9.5)	3 (10.3)	3 (8.8)	
No	53 (84.1)	25 (86.2)	28 (82.3)	
Unsure	4 (6.3)	1 (3.5)	3 (8.8)	
**Prior STI treatment**				0.297
Yes	26 (41.3)	14 (48.3)	12 (35.3)	
No	37 (58.7)	15 (51.7)	22 (64.7)	
**Sexually active** (in past 1 M)				**0.006** ^**c**^
Yes	48 (76.2)	27 (93.1)	21 (61.8)	
No	15 (23.8)	2 (6.9)	13 (38.2)	
**Illicit drug use** (in past 6 M)				0.326^c^
Yes	4 (6.3)	3 (10.3)	1 (2.9)	
No	59 (93.6)	26 (89.7)	33 (97.1)	
**HIV** (antibody on RDT)				0.080
Positive	27 (42.9)	9 (31.0)	18 (52.9)	
Negative	36 (57.1)	20 (69.0)	16 (47.1)	
**Syphilis** (treponemal antibody on RDT)				0.999^c^
Positive	6 (9.5)	3 (10.3)	3 (8.8)	
Negative	57 (90.5)	26 (89.7)	31 (91.2)	
**Ng NAAT**				0.694^c^
Positive	7 (11.1)	4 (13.8)	3 (8.8)	
Negative	56 (88.9)	25 (86.2)	31 (91.2)	
**Ct NAAT**				0.999^c^
Positive	6 (9.5)	3 (10.3)	3 (8.8)	
Negative	57 (90.5)	26 (89.7)	31 (91.2)	
Any curable STI*				0.712
Yes	16 (25.4)	8 (27.6)	8 (23.5)	
No	47 (74.6)	21 (72.4)	26 (76.5)	

P-value^a^ by rank sum test, P-value by chi-square, P-value^c^ Fishers exact test, ^b^only one participant had dual contraception (condoms and SDI), M – months, DMPA- Depot Medroxyprogesterone Acetate, IUD- Intrauterine device, RDT – rapid diagnostic test, Ng Neisseria gonorrhoeae, Ct Chlamydia trachomatis

*Any of syphilis, Ng, Ct

Similar proportions were either married or cohabiting 49.2% (31 of 63) or single, never married, separated, divorced, or widowed 50.8% (32 of 63). Most (69.8%) were employed; 28.6% (18 of 63) had engaged in transactional sex in the previous 6 months. The majority (84.1%) had never been tested for STIs, including HIV. Three quarters (76.2%) reported sex in the previous month. HIV prevalence was 36/92(39.1%); 10(27.8%) were new HIV diagnoses. Twenty-three participants had a curable STI; syphilis, gonorrhea, or chlamydia; 9/92(9.8%) each. Of those with negative HIV tests, 5/56(8.9%) reported current use of HIV pre-exposure prophylaxis (PrEP).

### Prevalence of family planning uptake

None of the 29 male participants reported condom use or vasectomy. Of the women, 46.0% (29 of 63) reported current FP use; data on the full study population can be found in Supplementary Table 1. Among women who used FP, the SDI was reported in 51.7% (15 of 29). More than one quarter, 27.6% (8 of 29), reported DMPA use. In contrast, condoms and IUDs were the least used methods, each reported by 10.3% (3 of 29) of women. Notably, only one participant reported dual methods, specifically condoms and SDI. No women reported the use of emergency contraception, oral contraceptives, or tubal ligation. 

Among female participants who reported transactional sex (*n* = 18), 14(77.8%) reported FP use; of these 3(21.4%) reported condom use, 3(21.4%) injectables, and 8(57.1%) SDIs. Among female participants with HIV (*n* = 27), 9(33.3%) reported FP use; 3(33.3%) reported using condoms, 2(22.2%) injectables, and 4(44.4%) SDIs.

### Factors that were significantly associated with family planning uptake

FP use in women was significantly associated with younger median age, being married/cohabiting, sex work, ≥ 2 sex partners in the past 3 months, and sexual activity in the past month. There was no significant association between FP use and HIV or curable STI status or previous STI testing and treatment (Table 1).

## Discussion

Among patients with GUD with a high prevalence of HIV, FP use was low in women and absent in men. Women’s FP use is consistent with other studies; in a nationally representative sample of 9,235 Ugandan women of reproductive age (15–49 years), 53.19% reported FP use [20]; in a study in Butaleja in eastern Uganda, 34% of women reported FP use [21]. Modern contraceptive use in SSA between 1995 and 2020 was 22%, with SDI reported as the most common [22], consistent with our findings. Another study conducted in Gulu City, Uganda, also reported the SDI as the most common method at 52.4% [23], possibly reflecting preference for discreet, effective methods that do not require daily adherence, or differences in availability of long-acting reversible FP methods. African men’s involvement in FP was low as in other studies [24, 25], and has been attributed to limited knowledge, social stigma, and patriarchal notions and norms [26].

Various studies highlight barriers to FP uptake at the individual level, such as fear of side effects [27, 28] lack of knowledge about FP, and misconceptions [29]. Community-level factors such as discouragement from relatives and close friends [29], and health facility-level factors such as unavailability of the preferred method and absence of trained personnel [30].

Despite the enhanced risk of HIV acquisition in people with GUD, less than 10% of HIV-uninfected participants reported HIV PrEP use. Inadequate knowledge about PrEP, including concerns related to side effects and stigma, may both limit uptake and adherence [31], suggesting overall limitations in sexual health education. Only one participant reported dual contraception; other studies reveal modest use of dual contraception. In a cross-sectional study conducted among female sex workers (FSW) in urban Uganda, dual contraception use was 58% [32]. In a northern Ugandan study, dual contraception was reported in 45% [33]. Taken together, these studies suggest that Ugandan women attending clinical services and those residing in urban slums might not be adequately informed about the benefits of dual contraception.

Younger age was significantly associated with FP use, consistent with several studies among Ugandan and Zambian women, where increasing age was inversely associated with modern FP use [34, 35]. Likewise, in high fertility countries in SSA, younger women were more likely to use FP [36]. A cross-sectional study among postpartum women in western Uganda reported that younger mothers were more likely to use postpartum FP [37]. In an urban setting, Ugandan women aged 40–49 years reported lower contraceptive uptake compared to those aged 15–24 years [38]. Younger age is associated with higher fertility [39], which may make younger women more vigilant about FP.

Being married or cohabiting was associated with FP use. This was also highlighted in a Tanzanian study among women of reproductive age [40]. Spousal communication and partner support in longer-term relationships may facilitate decision making and uptake of FP.

There were two predictable positive associations with FP use: sex work and recent sexual activity. One-third of the female participants reported transactional sex in the previous 6 months; most of these women reported FP use, possibly reflecting increased concern about unplanned pregnancies due to the nature of sexual encounters. In northern Uganda, 48.6% of the FSW interviewed reported at least one unintended pregnancy during sex work [41]. A study across SSA revealed that only 19% of FSW were planning a pregnancy [42]. Similarly, we found that having two or more sexual partners was significantly associated with FP use. Heightened unintended pregnancy and STI risks may explain the higher use of FP in this group of women. Higher reported use of FP in participants who reported sex in the past month is consistent with the results of another Ugandan study where women cited not being sexually active as their reason for not using contraceptives [38].

In our study, one-third of female participants with HIV reported FP use, similar to another study conducted in urban Uganda, where the uptake of FP was 38.4% among women with HIV on ART [38]. A systematic review and meta-analysis among women of reproductive age with HIV in SSA found that the pooled prevalence of unmet FP need was 30.1% [43], while globally, 14.9% of women of reproductive age in the general population had an unmet need [6]. Potential explanations for the low FP uptake in women with HIV in our study include a high prevalence of undiagnosed HIV and lack of FP services integrated into HIV care [44].

Our study has several strengths, including describing FP use in women with GUD who are at high risk of vertically transmitting STIs and may greatly benefit from effective FP. It also has limitations; the study was conducted among people with GUD and therefore cannot be generalized to other populations in Uganda. Other natural methods of FP, like lactational amenorrhea and the standard days method, were not assessed; this may have underestimated FP use. There was lack of information on reasons for non-use and potential confusion between condom use for contraception or HIV/STI prevention, which could have impacted the reliability of findings on condom use as a family planning method. Men were not asked about their partners’ FP use, which may have underestimated adequate FP within their relationships. Conversely, social desirability bias may have caused under- or overestimation of FP uptake; this was mitigated by asking personal questions at the end of the interview after establishing adequate rapport with the participants. The study sample was small and precluded multivariate analysis due to the low case number per event in relevant subgroups.

## Conclusion

Pregnancy was common in women with GUD. That two-thirds were enrolled outside of clinical settings suggests barriers to healthcare access. Despite clear behavioral risks in a setting where HIV/STI prevalence was high, FP uptake remained low, but consistent with other Ugandan studies. These findings underscore the need to strengthen sexual health services, promote FP education to increase uptake of FP overall, and encourage male condom use to prevent pregnancies and STIs. Community outreaches should educate the public and health care workers about GUD-associated risks and the benefits of effective FP in reducing mother-to-child transmission of STIs.

## Supplementary Information

Below is the link to the electronic supplementary material.

Supplementary Material 1

## Data Availability

Data is available from the senior author upon reasonable request.

## Abbreviations

ART: Antiretroviral Therapy
DMPA: Depot Medroxyprogesterone Acetate
FP: Family Planning
GUD: Genital Ulcer Disease
HIV: Human Immunodeficiency Virus
IDI: Infectious Diseases Institute
IQR: Interquartile Range
IUD: Intrauterine Device
NAATs: Nucleic Acid Amplification Tests
PrEP: Pre–Exposure Prophylaxis
RDT: Rapid Diagnostic Test
REC: Research Ethics Committee
SDI: Subdermal Implant
SSA: Sub–Sahara Africa
STIs: Sexually Transmitted Infections
TFR: Total Fertility Rate
USA: United States of America
VHT: Village Health Team
