# Supplementary material for: Uptake of family planning methods among adults presenting with genital ulcers at community posts and public outpatient health facilities in central Uganda
Source: Contracept Reprod Med. 2026 May 9;11:36. doi: 10.1186/s40834-026-00456-w (PMC13196166; doi:10.1186/s40834-026-00456-w)
Supplement: Supplementary file 1 — Supplementary Material 1 [file 40834_2026_456_MOESM1_ESM.docx]

**Supplementary Table 1: Baseline characteristics and Family Planning use among all participants with Genital Ulcer Disease**

| **Variable** | **All**  **n=92 (100.0)**  **n(col%)** | **Family planning use** | | **P value** |
| --- | --- | --- | --- | --- |
|  |  | **Yes**  **n=29 (31.5)**  **n (col%)** | **No**  **n=63 (68.5)**  **n (col%)** |  |
| **Age** (years) Median (IQR) | 30 (24-37.5) | 26 (22-30) | 33 (26-39) | **0.002**^a^ |
| **Age range** (years) |  |  |  | **0.012** |
| ≤24 | 26 (28.3) | 14 (48.3) | 12 (19.1) |  |
| 25-34 | 34 (37.0) | 9 (31.0) | 25 (39.7) |  |
| ≥35 | 32 (34.8) | 6 (20.7) | 26 (41.3) |  |
| **Gender** |  |  |  | **<0.001** |
| Male | 29 (31.5) | 0 | 29 (46.0) |  |
| Female | 63 (68.5) | 29 (100.0) | 34 (54.0) |  |
| **Marital status** |  |  |  | 0.067* |
| Single, never married | 25 (27.2) | 11 (37.9) | 14 (22.2) |  |
| Married, cohabiting | 45 (48.9) | 15 (51.7) | 30 (47.6) |  |
| Separate, divorced or widowed | 22 (23.9) | 3 (10.3) | 19 (30.2) |  |
| **Study recruitment site** |  |  |  | 0.188 |
| Clinic | 31 (33.7) | 7 (24.1) | 24 (38.1) |  |
| Community | 61 (66.3) | 22 (75.9) | 39 (61.9) |  |
| **Highest level of education** |  |  |  | 0.941* |
| <Primary seven | 40 (43.5) | 12 (41.4) | 28 (44.4) |  |
| ≥Primary seven | 45 (48.9) | 15 (51.7) | 30 (47.6) |  |
| None | 7 (7.6) | 2 (6.9) | 5 (7.9) |  |
| **Employment** |  |  |  | **0.011** |
| Employed | 72 (78.3) | 18 (62.1) | 54 (85.7) |  |
| Unemployed | 20 (21.7) | 11 (37.9) | 9 (14.3) |  |
| **Family planning method used^b^** |  |  |  | **na** |
| Male condom | 3 (10.3) | 3 (10.3) | na |  |
| Injections (DMPA) | 8 (27.6) | 8 (27.6) | na |  |
| Subdermal implant (SDI) | 15 (51.7) | 15 (51.7) | na |  |
| Coil or IUD | 3 (10.3) | 3 (10.3) | na |  |
| **Transactional sex** (in past 6 M) |  |  |  | **0.030** |
| Yes | 30 (32.6) | 14 (48.3) | 16 (25.4) |  |
| No^∞^ | 62 (67.4) | 15 (51.7) | 47 (74.6) |  |
| **Number sexual partners** (in past 3 M) |  |  |  | 0.075* |
| None | 9 (9.8) | 0 (0%) | 9 (14.3) |  |
| 1 | 49 (53.3) | 16 (55.2) | 33 (52.4) |  |
| ≥2 | 34 (37.0) | 13 (44.8) | 21 (33.3) |  |
| **Alcohol use** (in past 3 M) |  |  |  | 0.187 |
| Yes | 41 (44.6) | 10 (34.5) | 31 (49.2) |  |
| No | 51 (55.4) | 19 (65.5) | 32 (50.8) |  |
| **Illicit drug use** (in past 6 M) |  |  |  | 1.000* |
| Yes | 11 (12.0) | 3 (10.3) | 8 (12.7) |  |
| No | 81 (88.0) | 26 (89.3) | 55 (87.3) |  |
| **Previously tested for STI including HIV** |  |  |  | 0.057* |
| Yes | 23 (25.0) | 3 (10.3) | 20 (31.8) |  |
| No | 65 (70.6) | 25 (86.2) | 40 (63.5) |  |
| Unsure | 4 (4.4) | 1 (3.5) | 3 (4.8) |  |
| **Prior STI treatment** |  |  |  | 0.065 |
| Yes | 32 (34.7) | 14 (48.3) | 18 (28.6) |  |
| No | 60 (65.2) | 15 (51.7) | 45 (71.4) |  |
| **Sexually active** (in past 1 M) |  |  |  | **0.009*** |
| Yes | 70 (76.1) | 27 (93.1) | 43 (68.3) |  |
| No | 22 (23.9) | 2 (6.9) | 20 (31.7) |  |
| **HIV RDT** |  |  |  | 0.280 |
| Positive^ | 36 (39.1) | 9 (31.0) | 27 (42.9) |  |
| Negative | 56 (60.9) | 20 (69.0) | 36 (57.1) |  |
| **Syphilis RDT** |  |  |  | 1.000* |
| Positive | 9 (9.8) | 3 (10.3) | 6 (9.5) |  |
| Negative | 83 (90.2) | 26 (89.7) | 57 (90.5) |  |
| ***N. gonorrhoeae* NAAT** |  |  |  | 0.456* |
| Positive | 9 (9.8) | 4 (13.8) | 5 (7.9) |  |
| Negative | 83 (90.2) | 25 (86.2) | 58 (92.1) |  |
| ***C. trachomatis* NAAT** |  |  |  | 1.000* |
| Positive | 9 (9.8) | 3 (10.3) | 6 (9.5) |  |
| Negative | 83 (90.2) | 26 (89.7) | 57 (90.5) |  |
| **Any curable STI*** |  |  |  | 0.698 |
| Yes | 23 (25.0) | 8 (27.6) | 15 (23.8) |  |
| No | 69 (75.0) | 21 (72.4) | 48 (76.2) |  |

*P-value^a^ by rank sum test, P-value by chi-square, P-value^*^ Fishers exact test, ^b^only one participant had dual contraception (condoms and SDI) ^10 of 36(28%) were new HIV diagnoses. M – months DMPA- Depot Medroxyprogesterone Acetate, IUD- Intrauterine device, Curable STI*- syphilis, chlamydia or gonorrhea. ^∞^45 females reported no transactional sex.*
